# Network Rewiring in the Aging Immune System: From Chronic Inflammation to Age-Related Pathologies

**DOI:** 10.3390/cells15050414

**Published:** 2026-02-27

**Authors:** Ludmila Müller, Svetlana Di Benedetto

**Affiliations:** Max Planck Institute for Human Development, Lentzeallee 94, 14195 Berlin, Germany

**Keywords:** immunosenescence, inflammaging, age-related diseases, pro-resolving lipid mediators, impaired resolution of inflammation, systems-level and network-based perspective, therapy

## Abstract

Aging is accompanied by profound alterations in immune function that collectively drive increased susceptibility to infection, reduced vaccine efficacy, impaired tissue repair, and heightened risk of age-related diseases (ARDs). These alterations are characterized by the coexistence of immunosenescence and inflammaging. Rather than reflecting isolated cellular defects, immune aging emerges as a systems-level reprogramming of immune networks that disrupts the initiation, resolution, and regenerative phases of inflammatory responses. In particular, aging is associated with impaired resolution of inflammation, defective efferocytosis, reduced responsiveness to pro-resolving signals, and diminished regenerative capacity, leading to persistent inflammatory milieus and tissue damage. This review summarizes recent advances in the mechanisms underlying immune dysfunction in aging, with a focus on how chronic inflammation, failed resolution, and defective repair reinforce one another. We discuss how alterations in innate and adaptive immunity, immunometabolism, cellular senescence, and immune–tissue interactions drive inflammaging and contribute to major ARDs, including cancer, neurodegenerative, and cardiometabolic diseases. Finally, we highlight emerging therapeutic strategies aimed at restoring immune balance and resolution. By adopting a systems-level and network-based perspective, this review underscores immune aging as a modifiable driver of ARDs and identifies key knowledge gaps and future directions toward interventions that promote healthy aging and extended healthspan.

## 1. Introduction

Aging is associated with profound and progressive changes in immune system composition and function that affect both innate and adaptive immunity. This process, commonly referred to as immunosenescence, encompasses reduced immune responsiveness, impaired host defense against infections, diminished vaccine efficacy, and increased cancer incidence. Concomitantly, aging is characterized by a chronic, low-grade inflammatory state termed inflammaging, which persists in the absence of overt infection and contributes to functional decline across multiple tissues [1,2,3,4,5,6]. Together, immunosenescence and inflammaging represent interconnected hallmarks of immune aging that critically influence susceptibility to ARDs.

Accumulating evidence indicates that immune dysfunction is not merely a passive consequence of aging but a central driver of ARD pathogenesis, including cancer, neurodegenerative disorders, cardiovascular disease, and metabolic syndromes [4,7,8,9,10]. Aging-associated immune remodeling leads to altered leukocyte composition, impaired immune regulation, and sustained production of pro-inflammatory mediators [2,11]. Importantly, aging does not simply exaggerate inflammatory responses; rather, it fundamentally alters their kinetics and resolution. In young organisms, inflammation is typically transient and followed by an active resolution phase that restores tissue homeostasis. In contrast, aging disrupts this tightly regulated sequence, favoring persistent inflammation and tissue damage [12,13,14,15].

Resolution of inflammation is now recognized as an active and highly coordinated process involving specialized pro-resolving lipid mediators (SPMs), effective clearance of apoptotic cells through efferocytosis, and reprogramming of immune cells toward anti-inflammatory and reparative phenotypes [16,17]. Multiple components of these resolution pathways are compromised with age. Reduced biosynthesis of SPMs, impaired responsiveness to pro-resolving signals, and defective macrophage efferocytosis have been documented in aged tissues and experimental models [18,19,20]. These defects result in delayed termination of inflammatory responses and promote a sustained inflammatory milieu that predisposes tissues to chronic injury, fibrosis, and functional decline.

In parallel, immune aging interferes with regenerative and repair processes that are essential for maintaining tissue integrity. Immune cells play critical roles in coordinating regeneration by regulating stem- and progenitor-cell activation, angiogenesis, and extracellular matrix remodeling [12,21,22]. However, aging-associated changes in immune cell phenotype and plasticity shift the balance from regenerative to maladaptive repair responses, often favoring fibrosis over functional tissue restoration [23,24]. The convergence of impaired inflammatory resolution and defective regeneration therefore represents a key mechanistic link between immune aging and the progression of ARDs.

Despite increasing recognition of immune dysfunction as a fundamental hallmark of aging, therapeutic strategies to effectively modulate immune aging remain limited. Most current interventions aim to broadly suppress inflammation, with variable efficacy and potential adverse effects [25]. Emerging evidence suggests that approaches targeting the restoration of inflammatory resolution and regenerative immune functions—rather than indiscriminate immunosuppression—may offer more precise and durable benefits for healthy aging [8,26,27].

In this review, we summarize recent advances in the understanding of immune dysfunction during aging, with a particular focus on the mechanisms underlying impaired resolution, persistent inflammation, and defective regeneration. The review concentrates on chronic inflammatory and degenerative ARDs—including cancer, neurodegenerative, and cardiometabolic disorders—and their shared immune mechanisms, rather than on infection-specific or vaccinology-centered aspects of immune aging. Accordingly, the present review adopts a systems-level perspective on immune aging with emphasis on chronic inflammatory and degenerative ARDs, shared immune mechanisms, and emerging therapeutic strategies targeting resolution, regeneration, and immune–metabolic dysfunction.

In the context of this review, “network rewiring” refers to measurable alterations in the structure and function of immune interaction networks that occur during aging. These changes include shifts in immune cell composition and communication patterns, altered cytokine and signaling connectivity, dysregulated immune–metabolic integration, and disruption of resolution and regenerative feedback circuits. Rather than representing isolated defects, these coordinated alterations reshape immune system behavior at a systems level, promoting persistent inflammatory activation, impaired resolution, and maladaptive tissue responses that contribute to the development and progression of age-related diseases.

## 2. Hallmarks of Immune Dysfunction in Aging

**Figure 1:**
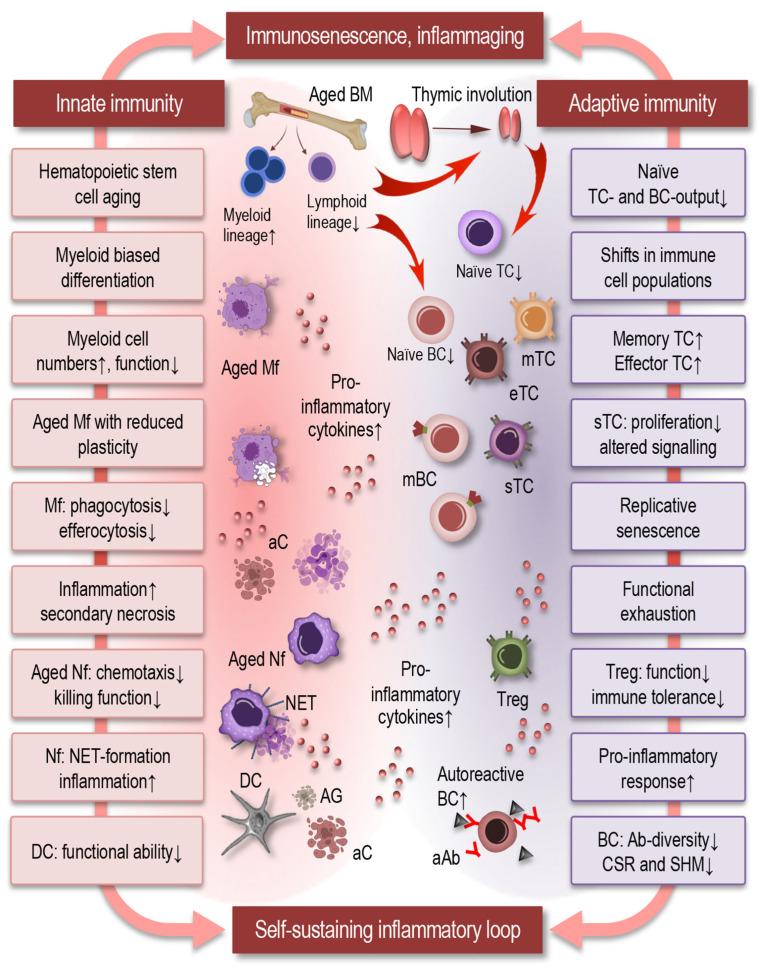
Hallmarks of immune dysfunction in aging. Immune aging is characterized by coordinated yet heterogeneous alterations across innate (**left**) and adaptive (**right**) immune compartments. Rather than a uniform decline, aging drives extensive remodeling of immune cell composition, phenotype, and function, resulting in reduced immune resilience and dysregulated inflammatory responses. These changes promote impaired resolution and defective repair, establishing self-sustaining inflammatory loops that contribute to chronic inflammation and age-related pathology. Abbreviations: BM: bone marrow; TC: T cell; mTC: memory T cell; eTC: effector T cell; sTC: senescent T cell; BC: B cell; mBC: memory BC; aC: apoptotic cell; Mf: macrophages; Nf: neutrophil; NET: neutrophil extracellular trap; Treg: regulatory T cell; DC: dendritic cell; AG: antigen; aAb: autoreactive antibody; CSR: class-switch recombination; SHM: somatic hypermutation.

Immune aging is characterized by coordinated yet heterogeneous changes affecting both innate and adaptive immune compartments (Figure 1). Rather than a uniform decline, aging induces a profound remodeling of immune cell composition, phenotype, and function, leading to reduced immune resilience and dysregulated inflammatory responses. These alterations collectively underlie the increased susceptibility to infections, impaired vaccine responses, chronic inflammation, and heightened risk of ARD observed in older individuals.

### 2.1. Alterations in Innate Immunity

The innate immune system undergoes substantial functional and phenotypic changes with age (Figure 1, left). Hematopoietic stem cell (HSC) aging promotes a myeloid-biased differentiation program, resulting in increased production of monocytes and granulocytes at the expense of lymphoid progenitors [10,28,29]. While myeloid cell numbers may be preserved or even increased, their functional capacity is often compromised.

Aged macrophages display reduced plasticity and an altered activation spectrum, with impaired transitions between pro-inflammatory and pro-resolving phenotypes. Defects in phagocytosis and efferocytosis—the clearance of apoptotic cells—are consistently observed and contribute to prolonged inflammation and secondary necrosis [30,31]. In parallel, aged macrophages show dysregulated cytokine production, characterized by elevated basal levels of pro-inflammatory mediators and blunted responses to acute stimuli.

Neutrophil function is also markedly altered during aging. Although neutrophil counts are often maintained, aged neutrophils exhibit impaired chemotaxis, reduced microbial killing, and altered formation of neutrophil extracellular traps (NETs), which may further propagate tissue damage and inflammation [32]. Dendritic cells display reduced antigen uptake, impaired migration, and diminished capacity to prime naïve T cells, thereby weakening the bridge between innate and adaptive immunity [6,29,33,34,35,36].

Collectively, these innate immune alterations contribute to ineffective pathogen clearance, persistent inflammatory signaling, and impaired orchestration of downstream immune responses.

### 2.2. Remodeling of Adaptive Immunity

Adaptive immune aging is dominated by a progressive loss of diversity and flexibility. Thymic involution, which begins early in adulthood and accelerates with age, leads to a marked reduction in naïve T-cell output (Figure 1, right). As a consequence, the peripheral T-cell pool becomes increasingly dominated by memory and highly differentiated effector T cells, many of which exhibit features of replicative senescence or functional exhaustion [1,11,27,37,38].

Senescent T cells display reduced proliferative capacity, altered signal transduction, and skewed cytokine profiles, often favoring pro-inflammatory responses [6]. Accumulation of late-differentiated CD8^+^ T cells with limited antigen specificity has been linked to impaired immune surveillance and increased susceptibility to viral infections and malignancies [39,40,41]. Regulatory T-cell function may also be altered with age, contributing to impaired immune tolerance and chronic inflammation [42].

B-cell aging is characterized by reduced generation of naïve B cells, diminished antibody diversity, and impaired class-switch recombination and somatic hypermutation [43,44,45]. These changes result in weaker and less durable humoral responses to infection and vaccination, alongside an increased propensity for autoantibody production. Together, adaptive immune remodeling leads to reduced responsiveness to new antigens while sustaining inflammatory activity driven by antigen-experienced cells.

The aging of the adaptive immune system reflects not only quantitative declines in lymphocyte populations but also qualitatively distinct senescence kinetics of the primary lymphoid organs. The thymus exhibits a well-characterized and rapid involution beginning after puberty and accelerating through adulthood [46,47,48,49]. In contrast, bone marrow lymphopoiesis exhibits a more gradual decline, driven by age-associated changes in HSCs and stromal niche alterations. Although B-cell progenitor numbers and function decrease with age, the kinetics of this decline are slower and less precipitous than thymic involution, and occur over a longer time course [47,48,50].

These differential aging trajectories result in disparate impacts on adaptive immunity: the thymus’s early and marked loss of function disproportionately diminishes naïve T-cell generation and diversity, whereas bone marrow alterations contribute to a gradual reduction in B-cell lineage output. The combination of these organ-specific declines helps to explain systemic consequences of immune aging, including reduced vaccine responsiveness, increased susceptibility to infections and malignancies, and the skewed balance between naïve and memory lymphocytes that underlies chronic immune dysregulation with age.

### 2.3. Systemic Consequences: Loss of Immune Homeostasis

The cumulative effects of innate and adaptive immune aging result in a systemic loss of immune homeostasis. Basal inflammatory tone is elevated, driven by senescent immune and non-immune cells, persistent innate immune activation, and defective resolution mechanisms [12,51]. This chronic inflammatory environment not only exacerbates tissue damage but also feeds back to further impair immune cell function, establishing self-sustaining inflammatory loops.

Importantly, immune aging is associated with reduced immune adaptability—the capacity to mount effective responses to acute challenges while efficiently terminating them [52]. This loss of flexibility underlies the paradoxical coexistence of immune deficiency and chronic inflammation in older individuals [25]. Rather than representing isolated defects, these hallmarks reflect a global reprogramming of immune networks that predisposes aging tissues to persistent inflammation, impaired repair, and progressive functional decline [53,54].

## 3. Inflammaging: Sources, Amplifiers, and Feedback Loops

**Figure 2:**
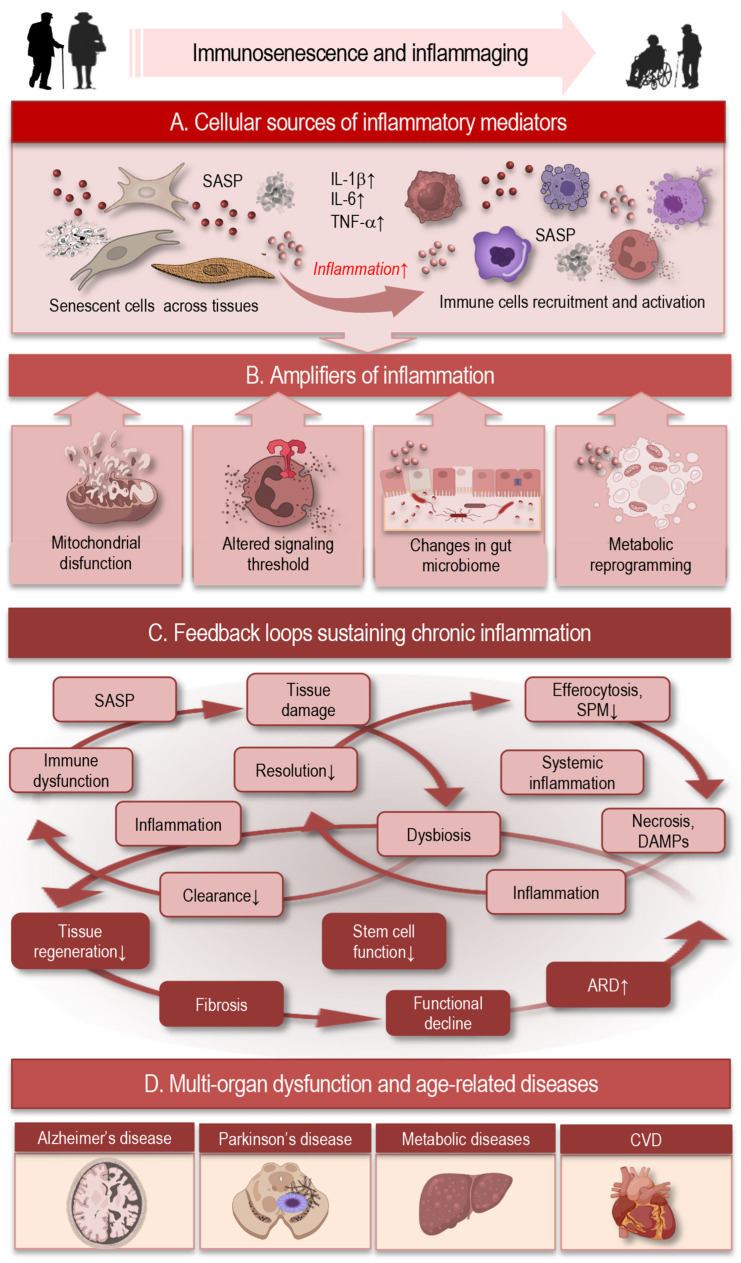
Inflammaging: sources and feedback loops. Inflammaging is a chronic, low-grade, age-associated inflammatory state driven by interconnected sources (**A**), amplifiers (**B**), and self-perpetuating feedback loops (**C**) that disrupt immune homeostasis. This persistent imbalance promotes the development of multiple ARDs (**D**), including cardiovascular, metabolic, and neurodegenerative disorders. Abbreviations: SASP: senescence-associated secretory phenotype; IL: interleukin; TNF: tumor necrosis factor; DAMP: danger-associated molecular pattern; SPM: pro-resolving lipid mediators; CVD: cardiovascular disease.

Inflammaging is defined as a chronic, systemic, low-grade inflammatory state that progressively increases with age and contributes to the pathogenesis of multiple ARDs including cardiovascular disease, metabolic syndrome, neurodegeneration, and frailty (Figure 2). While the term initially described an age-associated elevation in circulating inflammatory markers, it now encompasses a complex network of interlinked sources and amplifiers whose dynamic interactions create self-perpetuating feedback loops that sustain chronic inflammation and diminish immune homeostasis [55].

### 3.1. Cellular Sources of Inflammatory Mediators

A central contributor to inflammaging is the accumulation of senescent cells across tissues (Figure 2A). These cells adopt a senescence-associated secretory phenotype (SASP), releasing pro-inflammatory cytokines, including interleukin (IL)-6, IL-1β, tumor necrosis factor (TNF)-α, chemokines, growth factors, matrix metalloproteinases, and extracellular matrix fragments that alter local and systemic immune environments. SASP factors not only propagate inflammation locally but also recruit and activate immune cells, linking cellular senescence to chronic immune activation [13,53].

In addition to senescent stromal and epithelial cells, aged immune cells themselves acquire pro-inflammatory phenotypes. For example, age-related polarization shifts in macrophages favor sustained production of pro-inflammatory cytokines, and natural killer (NK) cells exhibit altered cytokine secretion and impaired cytotoxicity, further contributing to inflammatory load [56].

### 3.2. Amplifiers of Inflammation

Beyond primary inflammatory sources, inflammaging is sustained and intensified by multiple endogenous amplifiers that progressively shift immune signaling toward a chronically activated state (Figure 2B). A central amplifier is mitochondrial dysfunction, which emerges as a hallmark of aging across immune and non-immune cells. In aging, mitochondrial damage arises from converging hallmarks of cellular decline, including cumulative oxidative stress, impaired mitophagy and mitochondrial quality control, genomic instability, epigenetic drift, proteostatic failure, and metabolic dysregulation. These age-associated processes—closely linked to immunosenescence and inflammaging—are drivers of mitochondrial damage which amplify redox-sensitive signaling pathways and sustaining sterile inflammatory responses [57,58].

Age-associated mitochondrial damage leads to increased production of reactive oxygen species (ROS) and the cytosolic release of mitochondrial DNA (mtDNA), both of which act as potent danger-associated molecular patterns (DAMPs) [57,58,59].

These signals activate redox-sensitive transcriptional programs and innate immune sensors, including nuclear factor kappa-light-chain-enhancer of activated B cells (NF-κB), NLR family pyrin domain containing 3 (NLRP3) inflammasomes, and the cyclic GMP-AMP synthase (cGAS) and stimulator of interferon genes (STING)—cGAS–STING pathway, leading not only to NF-κB-dependent inflammatory gene expression but also to interferon regulatory factor 3 (IRF3) activation and induction of type-I interferon responses. Together, these pathways reinforce inflammatory signaling, interferon-stimulated gene expression, and cytokine release even in the absence of infection [59,60,61,62,63].

Chronic dysregulation of innate immune sensing further amplifies inflammaging. With age, pattern recognition receptors such as Toll-like receptors and cytosolic nucleic acid sensors exhibit heightened basal activity and impaired negative feedback control. Endogenous ligands derived from damaged cells, extracellular matrix breakdown, and protein aggregates continuously stimulate these receptors, maintaining a pro-inflammatory transcriptional baseline. This altered signaling threshold favors persistent cytokine production while simultaneously impairing responsiveness to acute immune challenges, thereby contributing to immune rigidity [64,65].

The aging gut microbiome represents another powerful amplifier of systemic inflammation. Age-related dysbiosis is characterized by reduced microbial diversity, loss of beneficial commensals, and expansion of pro-inflammatory taxa [66,67]. Concurrent deterioration of epithelial barrier integrity increases translocation of microbial products such as lipopolysaccharide into the circulation, resulting in tonic activation of innate immune pathways [53,68]. Recent human cohort and mechanistic studies demonstrate that gut-derived inflammatory signals strongly correlate with systemic inflammaging markers and predict frailty and multimorbidity in older adults [69,70].

Metabolic reprogramming of immune cells also plays a critical amplifying role. Aging is associated with shifts in immunometabolic pathways that favor sustained glycolysis, altered lipid handling, and impaired mitochondrial oxidative phosphorylation in myeloid cells [71]. These metabolic states support prolonged inflammatory cytokine production and reduce the capacity for phenotype switching toward anti-inflammatory or pro-resolving states. Importantly, metabolic dysfunction and inflammation are mutually reinforcing, creating a feed-forward loop that stabilizes inflammatory immune phenotypes [72,73].

Together, these amplifiers convert transient inflammatory stimuli into persistent inflammatory signaling, progressively locking immune cells and tissues into a pro-inflammatory state that resists resolution.

### 3.3. Feedback Loops Sustaining Chronic Inflammation

Inflammaging is ultimately maintained by interconnected feedback loops that link immune dysfunction, tissue damage, and impaired clearance mechanisms (Figure 2C). One of the most prominent loops involves cellular senescence and immune surveillance. Senescent cells accumulate with age and secrete a SASP, rich in inflammatory cytokines, chemokines, and matrix-remodeling enzymes. While senescent cells are normally cleared by immune mechanisms, aging compromises this clearance capacity. As a result, senescent cells persist, continue to release SASP factors, and further activate immune cells, reinforcing chronic inflammation and promoting the spread of senescence to neighboring cells [53,57].

A second major feedback loop operates at the level of inflammation resolution. Aging impairs key resolution pathways, including efferocytosis and the biosynthesis and signaling of specialized pro-resolving mediators. Defective clearance of apoptotic immune cells leads to secondary necrosis and release of additional DAMPs, which further stimulate innate immune receptors and prolong inflammation. This failure to properly terminate inflammatory responses transforms otherwise self-limiting immune reactions into chronic inflammatory states that progressively damage tissue architecture [30,31,74].

The microbiome–immune axis also forms a self-sustaining loop in aging. Chronic immune activation alters gut barrier function and microbial composition, while dysbiosis and microbial translocation further stimulate systemic inflammation. This reciprocal interaction perpetuates low-grade immune activation and contributes to sustained elevations of circulating inflammatory mediators, which in turn negatively affect distant organs, including the brain, vasculature, and musculoskeletal system [66,75,76,77,78]

At the systemic level, persistent inflammatory signaling interferes with tissue regeneration and repair, favoring fibrotic remodeling over functional restoration. Pro-inflammatory cytokines impair stem and progenitor cell function, disrupt extracellular matrix dynamics, and promote maladaptive wound-healing responses [79,80]. Over time, these processes may lead to progressive functional decline and increased vulnerability to age-related diseases, closing a final feedback loop in which inflammation both drives and is reinforced by tissue dysfunction [81,82].

Collectively, these feedback mechanisms demonstrate that inflammaging is not the result of isolated defects but reflects a network-level reprogramming of immune–tissue interactions. Once established, these loops stabilize chronic inflammation, erode immune adaptability, and accelerate biological aging. In metabolic tissues, for instance, inflammatory cytokines impair insulin signaling and lipid homeostasis, increasing the risk of type 2 diabetes [79,83].

### 3.4. Systemic and Organ-Level Consequences

Chronic low-grade inflammation, or inflammaging, exerts systemic effects that contribute to multi-organ dysfunction and age-related diseases (Figure 2D). Elevated circulating cytokines, including IL-6, TNF-α, and C-reactive protein (CRP), correlate with frailty, multimorbidity, and mortality in older adults [14,39,52]. These inflammatory signals accelerate biological aging, as measured by epigenetic clocks and other biomarkers of physiological dysregulation [84].

At the organ level, chronic inflammation drives structural and functional alterations. In the cardiovascular system, it promotes endothelial dysfunction and atherogenesis [85].

In metabolic tissues, inflammatory cytokines impair insulin signaling and lipid homeostasis, increasing the risk of type 2 diabetes [83,86]. In the brain, sustained neuroinflammation accelerates neurodegeneration and contributes to Alzheimer’s pathology [87,88]. Skeletal muscle and connective tissues are similarly affected: chronic inflammation disrupts regeneration, accelerates sarcopenia, and contributes to frailty [89,90].

Thus, across organ systems, persistent inflammatory signaling favors maladaptive repair, including fibrosis, which reduces tissue elasticity and functional reserve. This establishes a self-reinforcing cycle in which inflammation both drives organ decline and is amplified by tissue dysfunction. Inflammaging, therefore, represents a network-level perturbation linking immune aging to systemic and organ-specific pathophysiology, increasing vulnerability to cardiovascular, metabolic, neurodegenerative, and musculoskeletal disorders, and ultimately contributing to reduced lifespan.

## 4. Impaired Resolution of Inflammation in Aging

### 4.1. Resolution as an Active, Regulated Process

**Figure 3:**
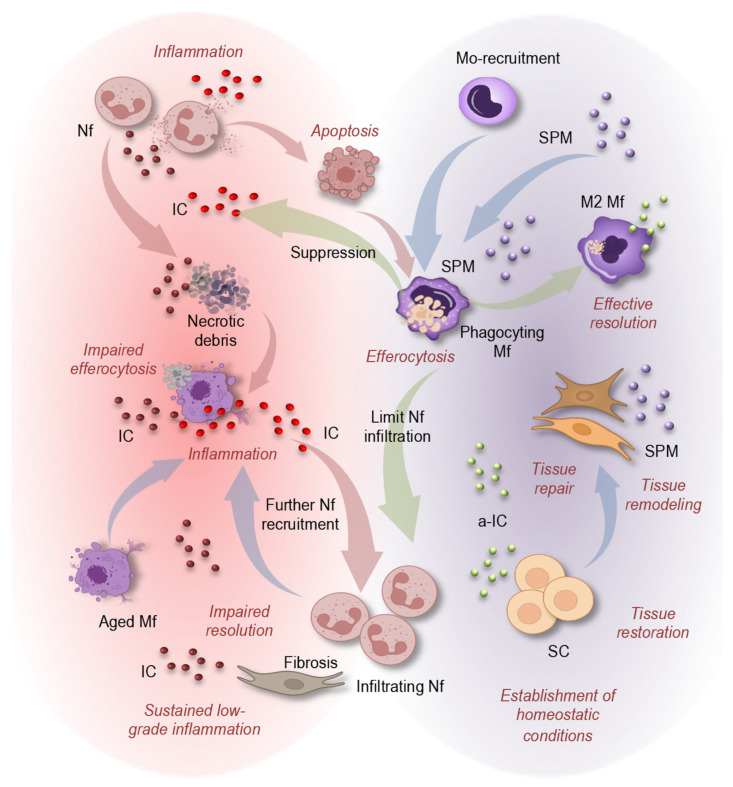
Inflammation and its impaired resolution during aging. The termination of inflammation is an active and tightly regulated biological process that is essential for restoring tissue homeostasis following immune activation. Inflammation is initiated by neutrophil (Nf) and monocyte (Mo) recruitment and inflammatory cytokine (IC) release, followed by neutrophil apoptosis or necrosis. Effective resolution (**right**) is driven by specialized pro-resolving mediators (SPM), which limit further neutrophil infiltration, promote monocyte (Mo) recruitment, and induce macrophage (Mf) efferocytosis and reprogramming toward reparative (M2) phenotypes, enabling tissue repair, remodeling, and restoration of homeostasis, including support of stem cell (SC)-mediated regeneration. In aging (**left**), macrophages display reduced phagocytic capacity, leading to impaired efferocytosis. Because efferocytosis promotes SPM production and suppresses inflammatory cytokines, its impairment creates a feed-forward loop that perpetuates inflammation. This results in defective resolution, persistent immune cell recruitment, fibrosis, accumulation of necrotic debris, and sustained low-grade inflammation, collectively contributing to inflammaging and tissue dysfunction.

The termination of inflammation is an active and tightly regulated biological process that is essential for restoring tissue homeostasis following immune activation (Figure 3). Resolution is not achieved through the passive decay of pro-inflammatory signals but instead requires the coordinated engagement of molecular and cellular programs that suppress further leukocyte recruitment, promote the clearance of inflammatory cells, and initiate tissue repair. This concept has fundamentally reshaped our understanding of inflammatory responses, establishing resolution as a distinct immunological phase governed by dedicated signaling pathways rather than as a mere endpoint of inflammation [91].

Central to this process is a lipid mediator class switch that favors the production of SPM, which actively counter-regulate inflammatory signaling, limit neutrophil infiltration, and promote macrophage reprogramming toward reparative phenotypes [92]. Through receptor-mediated mechanisms, SPM suppress pro-inflammatory gene expression while stimulating pathways linked to efferocytosis, tissue remodeling, and restoration of homeostasis [93]. In parallel, resolution signaling creates a permissive microenvironment for tissue repair by supporting stem and progenitor cell survival, activation, and regenerative capacity (Figure 3, right).

Beyond their role as anti-inflammatory signals, SPM function as active immunoresolvents that orchestrate temporal reprogramming of innate and adaptive immune responses. Derived enzymatically from ω-3 and ω-6 polyunsaturated fatty acids through lipoxygenase and cyclooxygenase pathways, resolvins, protectins, maresins, and lipoxins engage specific G-protein–coupled receptors such as formyl peptide receptor 2/lipoxin receptor (FPR2/ALX), chemerin receptor 23 (ChemR23), and G-protein-coupled receptor 32 (GPR32) to initiate transcriptional and metabolic programs distinct from classical anti-inflammatory signaling. These pathways suppress NF-κB–driven cytokine production while enhancing mitochondrial oxidative metabolism, cytoskeletal remodeling, and efferocytic capacity in macrophages. In parallel, SPM modulate neutrophil trafficking by reducing integrin activation and promoting apoptosis, thereby facilitating non-phlogistic clearance. Importantly, resolution programs also influence stromal and progenitor cell compartments by limiting oxidative stress and maintaining niche integrity, linking immune resolution directly to tissue regeneration and functional recovery [15,18,94,95]. Effective resolution therefore represents a distinct immunological state, genetically and metabolically programmed, that is as critical for tissue health as the initial inflammatory response itself.

### 4.2. Age-Related Defects in Resolution Pathways

Aging is associated with a progressive decline in the efficiency of resolution pathways, resulting in prolonged inflammatory responses and incomplete restoration of tissue homeostasis. Experimental models and human studies demonstrate that aged tissues exhibit a reduced capacity to generate SPM following inflammatory challenge, leading to delayed termination of leukocyte recruitment and extended inflammatory duration [31]. In parallel, aging alters receptor expression and downstream signaling responsiveness in immune cells, further blunting pro-resolving signaling.

One of the most consistent defects observed with aging is impaired efferocytosis by macrophages (Figure 3, left). Aged macrophages display reduced phagocytic capacity, defective cytoskeletal remodeling, and altered metabolic programming, all of which compromise efficient clearance of apoptotic inflammatory cells [96]. Because successful efferocytosis feeds back to enhance SPM production and suppress inflammatory cytokine release, its impairment creates a feed-forward loop that perpetuates inflammation.

Delayed clearance of neutrophils and monocytes further exacerbates these defects. Persisting inflammatory cells undergo secondary necrosis, releasing danger-associated molecular patterns that sustain innate immune activation and prevent the timely transition toward resolution. As a result, aging skews inflammatory responses toward a prolonged, low-grade but self-sustaining state characterized by resolution failure rather than exaggerated initiation [97].

Mechanistically, age-associated resolution defects arise from alterations in both SPM biosynthesis and receptor-mediated signaling networks. Aging modifies lipid metabolism through oxidative stress, mitochondrial dysfunction, and shifts in membrane fatty-acid composition, collectively reducing substrate availability and enzymatic efficiency required for SPM generation. Concurrently, aged macrophages and neutrophils exhibit dysregulated G-protein-coupled receptor (GPCR) signaling, impaired calcium flux, and altered downstream kinase activation, limiting responsiveness to pro-resolving cues [26,31,98].

Epigenetic remodeling and senescence-associated transcriptional programs further bias myeloid cells toward persistent inflammatory phenotypes, reducing their capacity for metabolic switching toward oxidative phosphorylation—a process required for efficient efferocytosis and tissue repair. In addition, aged stromal and endothelial cells display diminished production of resolution-supportive mediators and altered extracellular matrix composition, creating a microenvironment that favors leukocyte persistence rather than termination of inflammation. Together, these molecular and cellular alterations transform resolution from a coordinated termination program into a delayed and incomplete process that sustains chronic inflammatory signaling in aging tissues [26,64,81,99,100,101].

### 4.3. Consequences of Failed Resolution

The inability to effectively resolve inflammation has profound consequences for tissue integrity and organismal health. Persistent inflammatory infiltrates maintain elevated levels of cytokines, proteases, and reactive oxygen species that can disrupt normal tissue architecture and compromise organ function. In this context, inflammatory responses increasingly shift from adaptive host defense to maladaptive tissue injury [102].

Resolution failure can promote aberrant wound-healing programs characterized by sustained fibroblast activation, excessive extracellular matrix deposition, and progressive fibrosis (Figure 3, left). These processes have been documented across multiple organs, including the cardiovascular system, lung, liver, and kidney, where unresolved inflammation accelerates structural remodeling and functional decline [91]. Importantly, fibrotic remodeling can further impair tissue elasticity and regenerative capacity, increasing susceptibility to subsequent inflammatory insults.

At the systemic level, chronic resolution defects may contribute to persistent immune activation and reinforce inflammaging. Tissue damage resulting from unresolved inflammation generates additional danger signals that perpetuate immune stimulation, establishing a self-reinforcing cycle in which inflammation both drives and is amplified by tissue dysfunction. Impaired resolution therefore represents a central mechanistic link between immune aging, chronic inflammation, and the progression of ARD [26,103].

## 5. Defective Regeneration and Tissue Repair in the Aging Immune System

Effective tissue regeneration following injury relies on a tightly coordinated dialogue between immune cells, stromal compartments, and resident stem and progenitor cells. In young organisms, inflammatory responses are rapidly followed by resolution programs that actively promote repair through immune cell reprogramming, growth factor release, and extracellular matrix remodeling. With aging, this coordination becomes progressively disrupted, resulting in delayed or incomplete regeneration and a shift toward maladaptive repair outcomes [104,105].

Aging alters the functional plasticity of innate immune cells that orchestrate tissue repair. Macrophages, which normally transition from inflammatory to reparative phenotypes during resolution, exhibit impaired phenotypic switching in aged tissues, maintaining pro-inflammatory transcriptional programs while failing to adequately support angiogenesis, matrix remodeling, and progenitor cell activation [91,106]. This persistent inflammatory bias can interfere with the regenerative microenvironment required for effective tissue restoration.

In parallel, chronic exposure to inflammatory cytokines directly compromises stem and progenitor cell function. Experimental and human studies demonstrate that prolonged signaling through pathways such as TNF, IL-1, and IFN disrupts stem cell quiescence, reduces self-renewal capacity, and biases differentiation toward dysfunctional or senescent states [107,108]. An additional layer of complexity is introduced by extracellular vesicles (EVs), which transport cytokines and other signaling mediators between cells and thereby shape regenerative and inflammatory processes in aging tissues. EV-associated cytokines can exert context-dependent effects determined by their cellular origin and cargo composition, influencing stem and progenitor cell behavior within local niches [109,110,111,112,113].

Immune cell-derived EVs enriched in inflammatory mediators may propagate senescence-associated signals and impair tissue repair, whereas exercise-induced muscle-derived EVs carrying myokines can promote anti-inflammatory and pro-regenerative responses. In contrast, adipose-derived EVs contribute to chronic low-grade inflammation and metabolic dysregulation that negatively affect stem cell function, while microglial EVs can amplify neuroinflammatory signaling and hinder neural repair. EV-mediated cytokine delivery may alter signaling kinetics and cellular targeting compared to freely circulating cytokines, thereby modulating regenerative outcomes during aging [109,111,112,113,114,115].

Defective immune-mediated clearance mechanisms may also contribute to impaired repair. Inefficient removal of apoptotic cells and tissue debris sustains local danger signaling, reinforcing inflammatory activation and preventing the establishment of a pro-regenerative niche. As unresolved inflammation persists, fibroblasts and myofibroblasts are preferentially activated, promoting matrix deposition and fibrotic remodeling rather than functional tissue replacement [96,104,105].

These effects are further amplified by aging-associated changes in the extracellular matrix, where inflammatory remodeling may alter mechanical and biochemical cues essential for regeneration. Chronic immune activation promotes fibroblast and myofibroblast reprogramming, leading to excessive deposition of collagen, fibronectin, and cross-linked matrix components that can increase tissue stiffness and disrupt stem and progenitor cell niches [21,23,116]. In skeletal muscle, inflammatory macrophage–fibroblast crosstalk drives fibrotic ECM accumulation that impairs satellite cell activation and regenerative myogenesis [117].

In the lung and liver, persistent immune–stromal signaling promotes aberrant matrix remodeling that restricts epithelial and hepatocyte progenitor expansion and favors fibrosis over functional tissue repair [118,119]. Pathological ECM accumulation during liver aging creates a fibrotic niche in which mesenchymal cells, immune cells, and endothelial cells cooperate to promote scar formation rather than functional regeneration. In this context, macrophages and other immune cells can drive profibrotic signaling and matrix deposition, resulting in persistent fibrosis and impaired hepatocyte regenerative responses [119,120].

Similarly, in the aging bone marrow, inflammatory cytokines reshape the stromal and ECM landscape, altering hematopoietic stem cell adhesion, quiescence, and lineage commitment. Elevated niche rigidity can activate mechano-sensitive transcriptional regulators in stromal cells, while dysregulated ECM proteolysis generates bioactive fragments that interfere with HSC–stromal interactions [121,122,123]. These alterations in the bone marrow microenvironment favor myeloid skewing and reduce regenerative capacity with age. Collectively, these immune–stroma–ECM interactions create a microenvironment that perpetuates dysfunctional regeneration and reinforces chronic inflammatory signaling during aging.

At the systemic level, defective regeneration feeds back into immune aging. Tissue dysfunction and fibrosis generate persistent stress and damage signals that perpetuate immune activation, reinforcing inflammaging and further impairing regenerative responses. This establishes a self-sustaining loop in which immune dysfunction may limit repair, and failed repair in turn amplifies immune dysregulation. As a result, impaired tissue regeneration represents not only a consequence but also a driver of immune aging and age-related disease progression [124,125,126].

## 6. Immune Aging and the Pathophysiology of Age-Related Diseases

The chronic immune dysregulation and defective resolution described in the previous sections do not remain confined to isolated tissues. Instead, they propagate systemic alterations that may compromise multiple organs, creating a permissive environment for the development and progression of ARDs. Persistent low-grade inflammation, impaired efferocytosis, defective SPM signaling, and maladaptive tissue repair collectively generate a network of immune–tissue interactions that underlie many age-associated pathologies. By integrating these shared mechanisms, immune aging may emerge not only as a contributor but also as a central driver of ARDs, linking cellular and molecular defects to organ-level dysfunction and clinical disease. This section summarizes the current understanding of how immune aging contributes to the pathophysiology of key ARDs and highlights common immune mechanisms that underlie these disorders.

### 6.1. Cancer

Cancer exemplifies how systemic immune aging can reshape disease susceptibility through network-level alterations across both innate and adaptive compartments. Age-related remodeling of immune cell interactions, chronic inflammatory signaling, and impaired resolution may collectively create an environment in which tissue homeostasis is destabilized and oncogenic processes can progress unchecked. These systemic changes are not confined to local tumor microenvironments but reflect coordinated dysregulation across multiple organs and immune networks, linking cancer risk to broader patterns of age-related immune dysfunction and chronic inflammation.

**Figure 4:**
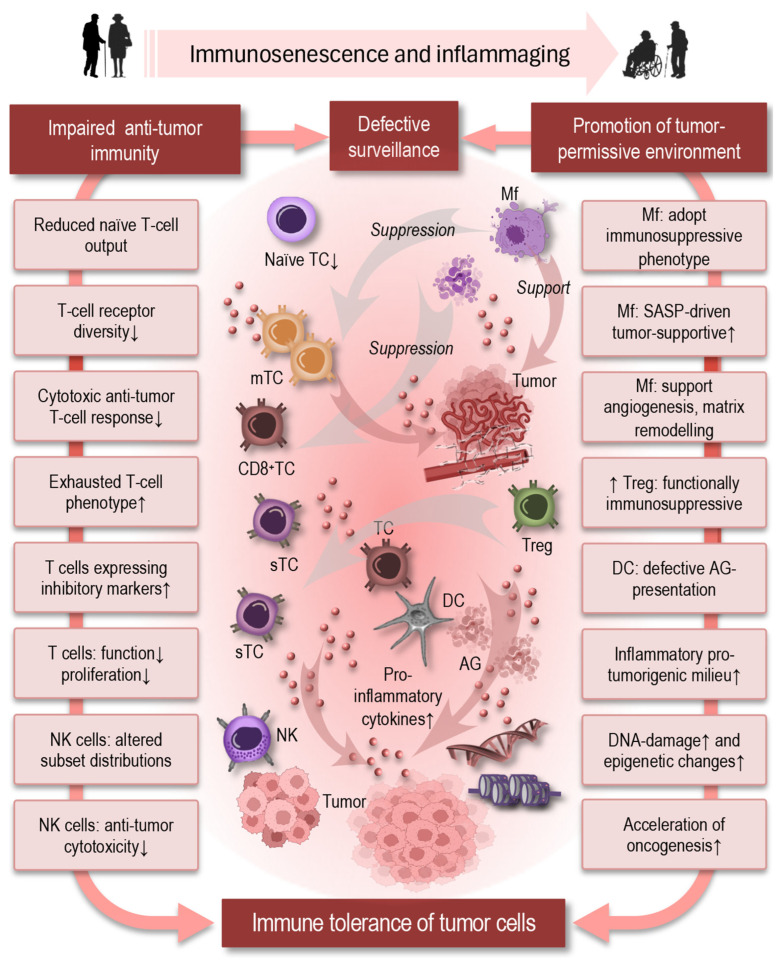
Immune aging compromises immune surveillance and promotes a tumor-permissive environment, leading to immune tolerance of malignant cells and accelerating oncogenesis. Abbreviations: TC: T cell; mTC: memory T cell; CD: cluster of differentiation; sTC: senescent T cell; Mf: macrophages; NK: natural killer cells; Treg: regulatory T cell; DC: dendritic cell; AG: antigen; DNA: deoxyribonucleic acid.

Immune surveillance plays a central role in controlling emerging neoplasms, yet immune aging may degrade this capacity (Figure 4). With advancing age, thymic involution reduces the output of naïve T cells and narrows T-cell receptor diversity, limiting the repertoire available to recognize diverse tumor antigens and diminishing effective cytotoxic T-cell responses against emerging neoplastic cells [127].

This is compounded by an increase in exhausted and senescent T-cell phenotypes within the peripheral pool, which express inhibitory markers and exhibit reduced proliferative and effector capacities, further impairing anti-tumor immunity [128].

Innate immune cells are also affected by aging in ways that promote a tumor-permissive environment. Natural killer cells, key effectors of early anti-tumor responses, display altered subset distributions and diminished cytotoxicity in older adults, undermining their ability to eliminate transformed cells [1,129]. Macrophages in aged tissues often adopt immunosuppressive or tumor-associated phenotypes, driven in part by chronic inflammatory cues and SASP, which can support angiogenesis, matrix remodeling, and tumor growth rather than tumor clearance. The accumulation of immunosuppressive regulatory T cells with age and age-related defects in dendritic-cell antigen presentation further compromise adaptive anti-tumor responses, creating a microenvironment that facilitates immune evasion and tumor progression [49,130].

Inflammaging contributes mechanistically to tumor development and progression by sustaining a pro-tumorigenic milieu. Chronic exposure to inflammatory cytokines such as IL-6, IL-1β, and TNF-α promotes cellular proliferation and survival signaling, stimulates angiogenesis, and can induce DNA damage and epigenetic alterations in non-malignant cells, accelerating oncogenesis [124,129,130,131]. The prevalence of low-grade inflammation in older adults thus converges with immunosenescence to tip the balance from effective surveillance toward immune tolerance of tumor cells [128,132,133].

### 6.2. Neurodegenerative Disorders

**Figure 5:**
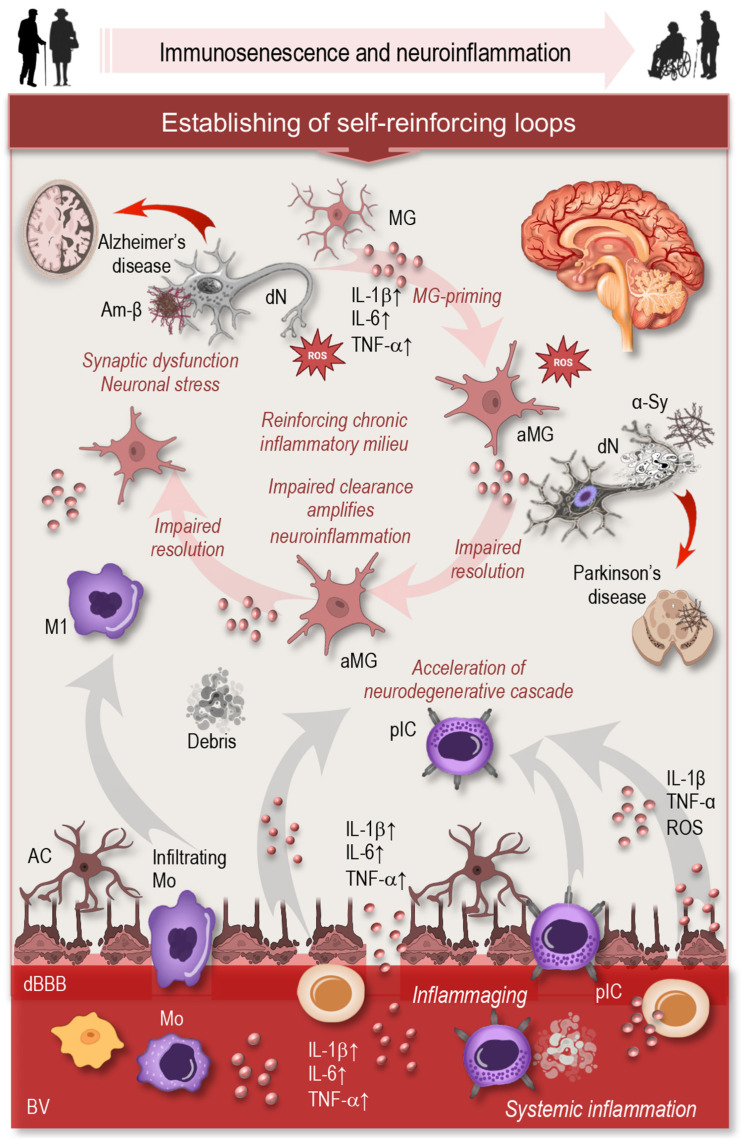
Aging profoundly alters central and peripheral immune responses, affecting brain homeostasis and resilience. Key clearance mechanisms decline, as microglial phagocytic capacity, lysosomal function, and metabolic efficiency decrease, impairing the removal of protein aggregates (amyloid-β, α-synuclein), apoptotic neurons, and synaptic debris. This amplifies neuroinflammation and promotes accumulation of toxic proteins that drive Alzheimer’s and Parkinson’s disease progression. Together, these changes create a self-reinforcing loop in which immune aging sustains chronic neuroinflammation, impaired clearance, and neuronal damage, while ongoing neurodegeneration generates additional danger signals that perpetuate immune activation. Abbreviations: MG: microglia; aMG: activated microglia; Am-β: amyloid-β; dN: degenerating neuron; IL: interleukin; TNF: tumor necrosis factor; α-Sy: α-synuclein; Mo: monocyte; M1: M1 macrophage; dBBB: disrupted blood–brain barrier; BV: blood vessel; pIC: peripheral immune cell.

Neurodegenerative disorders represent a major class of ARDs in which immunosenescence plays a critical and increasingly well-defined role (Figure 5). Aging is associated with profound changes in both central and peripheral immune responses that affect brain homeostasis, resilience, and vulnerability to neurodegeneration [12]. Microglia, the resident innate immune cells of the central nervous system, undergo age-associated priming characterized by heightened basal inflammatory tone, exaggerated responses to secondary stimuli, and impaired ability to return to homeostatic states [134]. This primed phenotype promotes sustained production of pro-inflammatory cytokines and reactive oxygen species, contributing to synaptic dysfunction and neuronal stress [12,73,134].

Aging also compromises key resolution and clearance mechanisms within the brain. Microglial phagocytic capacity declines with age, reducing efficient removal of protein aggregates such as amyloid-β and α-synuclein, as well as apoptotic neurons and synaptic debris. This decline is accompanied by altered microglial phenotypes and reduced lysosomal and metabolic efficiency, further limiting effective clearance. Impaired removal of cellular and proteinaceous debris amplifies neuroinflammation and facilitates the accumulation of toxic protein species that drive disease progression in Alzheimer’s and Parkinson’s disease [87,135]. In parallel, reduced responsiveness to pro-resolving signals further delays termination of inflammatory responses, reinforcing a chronic inflammatory milieu within neural tissues [18].

Peripheral immune aging contributes additional layers of dysregulation that intersect with CNS pathology. Increased systemic inflammation, altered monocyte phenotypes, and age-related disruption of blood–brain barrier integrity may enhance immune–brain crosstalk and permit greater infiltration of peripheral immune cells into the central nervous system. Once within neural tissues, these cells can adopt pro-inflammatory states, amplify local cytokine and chemokine signaling, and interact with resident microglia, thereby accelerating neurodegenerative cascades [12,73,136,137]. Moreover, senescent cells accumulating in the aging brain and periphery release inflammatory mediators that may further amplify microglial activation and neuronal vulnerability [12,138].

Collectively, these processes can establish a self-reinforcing loop in which immune aging promotes chronic neuroinflammation, impaired clearance, and neuronal damage, while ongoing neurodegeneration generates additional danger signals that perpetuate immune activation [73,138]. As in other ARDs, neurodegenerative disorders therefore emerge not solely from intrinsic neuronal defects but from progressive dysregulation of immune–tissue interactions that accompany aging, positioning immune aging as a central driver of disease onset and progression.

### 6.3. Cardiovascular and Metabolic Disorders

**Figure 6:**
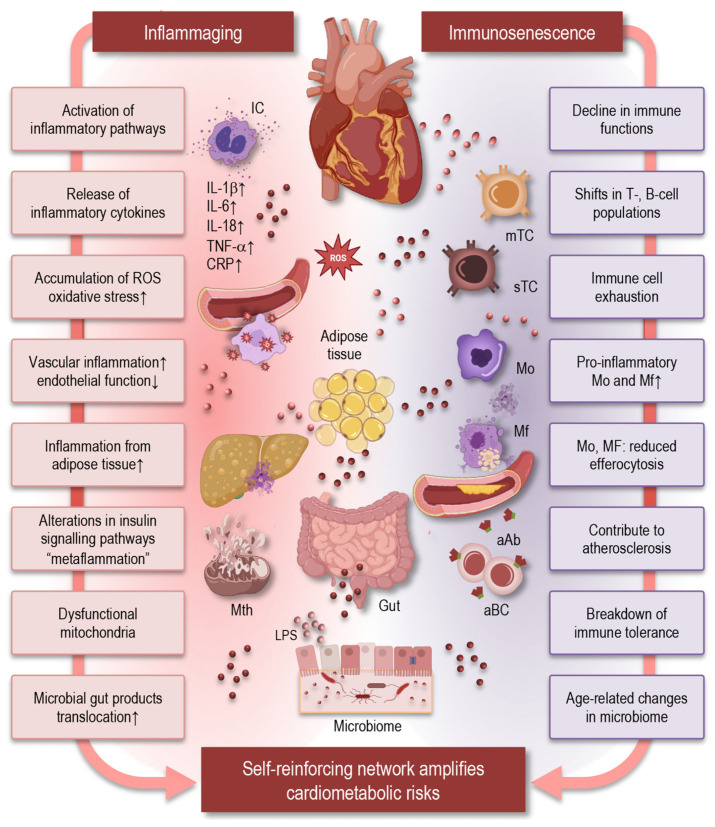
Immune aging drives cardiovascular and metabolic dysfunction through chronic inflammation, innate and adaptive immune dysregulation, and maladaptive tissue responses. Inflammaging-associated oxidative stress, mitochondrial dysfunction, and age-related gut dysbiosis amplify metabolic impairment and cardiometabolic risk. These processes can form a self-reinforcing network, in which chronic inflammation promotes tissue dysfunction and metabolic derangement, while tissue stress and altered metabolic signals sustain immune activation. Abbreviations: IC: immune cell; IL: interleukin; TNF: tumor necrosis factor; CRP: C-reactive protein; mTC: memory T cell; sTC: senescent T cell; Mo: monocyte; aBC: autoreactive B cells; aAb: autoreactive antibody; Mf: macrophages; Mth: mitochondria; LPS: lipopolysaccharide.

Cardiovascular and metabolic disorders are among the most prevalent and debilitating consequences of aging, and immune aging can contribute directly to their pathophysiology through shared mechanisms of chronic inflammation, innate and adaptive immune dysregulation, and maladaptive tissue responses (Figure 6). The interplay of inflammaging and immunosenescence creates a pro-inflammatory vascular milieu that drives endothelial dysfunction, vascular remodeling, and the progression of atherosclerosis, hypertension, and related cardiovascular diseases (CVDs) in older adults [139].

Age-associated chronic low-grade inflammation is characterized by elevated cytokines such as IL-6, IL-1β, and TNF-α, as well as high-sensitivity C-reactive protein (hsCRP), which collectively contribute to vascular injury, oxidative stress, and impaired endothelial homeostasis [10,139,140]. Monocyte and macrophage populations in aged individuals exhibit increased pro-inflammatory polarization and reduced capacity for efferocytosis, contributing to plaque progression and instability in atherosclerosis [10].

Dysfunctional T- and B-cell responses further compound vascular inflammation; aging skews T-cell subpopulations toward senescent and pro-inflammatory phenotypes and diminishes regulatory mechanisms that ordinarily restrain chronic inflammatory signaling in vascular tissues. Such adaptive immune changes may contribute both to sustained vascular inflammation and to the breakdown of immune tolerance, which can promote autoantibody production and maladaptive immune responses linked to plaque instability and cardiovascular events [141].

Metabolic disorders, including insulin resistance and type 2 diabetes, are closely tangled with immune aging. Chronic inflammatory signaling emanating from adipose tissue macrophages and senescent immune cells alters insulin signaling pathways and lipid metabolism, creating a state of “metaflammation” that predisposes older individuals to metabolic dysregulation [142]. Inflammaging-driven oxidative stress and mitochondrial dysfunction within metabolic tissues further exacerbate metabolic impairment and promote cardiometabolic risk [143]. Age-related changes in the gut microbiome and increased microbial translocation also contribute to systemic metabolic inflammation, linking gut immune interactions with metabolic and cardiovascular dysregulation [85].

Importantly, these mechanisms do not operate in isolation. The influence of immune aging on cardiovascular and metabolic disorders reflects a self-reinforcing network in which chronic inflammation may promote tissue dysfunction and metabolic derangement, and in turn, tissue stress and altered metabolic signals sustain immune activation [10]. This integrated perspective illustrates how immune aging can shape not only the risk of CVDs and metabolic syndromes but also their progression through shared immunological pathways such as persistent low-grade inflammation, impaired resolution of inflammation, and maladaptive immune–tissue interactions.

## 7. Therapeutic Strategies to Modulate Immune Aging

Therapeutic targeting of immune aging aims to rebalance dysregulated inflammatory networks, restore immune adaptability, and improve tissue repair capacity. Current approaches range from mechanistically targeted pharmacological agents to regenerative, metabolic, lifestyle, and precision strategies. Evidence strength varies considerably, with some interventions supported by early clinical data and others remaining primarily experimental. A critical evaluation of efficacy, safety, and translational readiness is therefore essential.

### 7.1. Targeting Core Inflammatory and Senescence Pathways

Interventions directed at fundamental drivers of immune aging, including chronic inflammatory signaling and cellular senescence, represent the most mechanistically advanced therapeutic class. Modulation of the mechanistic target of rapamycin (mTOR) pathway—through agents such as rapamycin and its analogs—has been shown to recalibrate immune metabolism, attenuate excessive inflammatory signaling, mitigate components of the SASP, and enhance antiviral responses in older adults, with early-phase clinical trials providing supportive evidence of immunological benefit [27,144,145]. However, potential risks include metabolic dysregulation, impaired wound healing, and dose-dependent immunosuppression, emphasizing the need for intermittent or low-dose regimens.

Senolytic and senomorphic therapies aim to reduce SASP-driven inflammatory amplification by eliminating or reprogramming senescent cells. Compounds such as dasatinib plus quercetin, fisetin, and the B-cell lymphoma 2 (BCL-2) inhibitors have shown promising preclinical effects on immune homeostasis and tissue inflammation. Nevertheless, clinical evidence remains preliminary, safety concerns stay significant, including thrombocytopenia, off-target toxicity, and uncertain long-term immune consequences. Clinical translation is still early, and patient stratification will likely be essential [146,147,148,149].

### 7.2. Modulating of Inflammatory Signaling Networks and Resolution Pathways

Targeting intracellular inflammatory signaling represents a complementary strategy to rebalance immune network activity. Inhibitors of p38 mitogen-activated protein kinase (p38 MAPK) can restore macrophage functionality, enhance efferocytosis, and promote pro-resolving phenotypes in aging models [150,151,152]. While mechanistically attractive, long-term systemic kinase inhibition may carry risks related to host defense impairment and unintended metabolic effects.

Nanoparticle-based platforms designed to enhance resolution and efferocytosis demonstrate growing promise, particularly in chronic inflammatory diseases such as atherosclerosis [153]. These approaches offer targeted delivery and reduced systemic toxicity, but clinical evidence remains limited and manufacturing complexity poses translational challenges.

### 7.3. Regenerative and Immune-Reconstitution Strategies

Cellular and regenerative interventions aim to restore immune architecture and adaptive capacity. Mesenchymal stem cells (MSCs)-based therapies exhibit immunomodulatory and tissue-repair properties, with encouraging preclinical and early clinical data suggesting benefits for inflammatory dysregulation and impaired regeneration. However, heterogeneity in cell preparations, uncertain durability of effects, and potential tumor-promoting signals remain key concerns [154,155,156,157].

Reconstitution of adaptive immune output through thymic and hematopoietic rejuvenation represents an emerging but strategically important avenue. Beyond IL-7 supplementation, several molecular regulators are under investigation. Forkhead box N1 (FOXN1)-associated pathways, keratinocyte growth factor (KGF), and fibroblast growth factor (FGF) 21 contribute to thymic epithelial integrity and naive T-cell production, with preclinical evidence indicating delayed thymic involution and improved immune function. Exercise and metabolic modulators such as metformin have demonstrated supportive effects on thymic metabolism and epithelial function in experimental systems, suggesting indirect but potentially scalable approaches. Additional strategies including sex steroid modulation, caloric restriction mimetics, and cytokine combinations are under evaluation. While conceptually compelling, most approaches remain at the preclinical or early translational stage, and long-term immune balance must be carefully monitored [25,53,158,159,160,161].

### 7.4. Lifestyle and Nutritional Interventions

Lifestyle-based strategies provide broad systemic benefits and represent the most immediately translatable interventions [53,162]. Regular physical activity, structured stress reduction, and adequate sleep correlate with reduced inflammatory cytokine levels, improved innate and adaptive immune responses, and enhanced vaccine responsiveness in aging populations [146,163,164,165,166]. Despite strong epidemiological support, effect sizes are generally modest and highly dependent on adherence and baseline health status.

Nutritional optimization, particularly correction of micronutrient deficiencies such as vitamins C, D, E, zinc, and selenium, can improve immune cell function and reduce chronic inflammation [53,167,168,169,170]. Omega-3 fatty acids serving as precursors for pro-resolving lipid mediators contribute to resolution pathways and may enhance inflammatory control [171,172,173,174,175]. However, heterogeneity across clinical trials and limited large-scale randomized evidence prevent definitive conclusions regarding long-term disease modification.

### 7.5. Microbiome-Targeted Strategies: Emerging Systems-Level Modulators

Modulation of the gut microbiome through dietary fiber, prebiotics, probiotics, and microbiome-directed therapies can influence systemic inflammation and immune regulation [176,177,178]. Diets rich in fiber and prebiotics, targeted probiotic supplementation, and microbiome-directed interventions can enhance gut barrier integrity, promote beneficial microbial taxa, and reduce translocation-induced inflammaging, thereby influencing systemic immune function and inflammatory set points. Improvements in barrier integrity and microbial metabolite production may reduce translocation-driven inflammatory activation. While mechanistically promising and supported by observational studies, variability between individuals and limited standardized clinical trials currently restrict therapeutic generalization [53,179,180].

### 7.6. Precision and Multi-Modal Interventions: Future Direction

Emerging personalized strategies integrating immunoprofiling, metabolomics, and epigenetic data aim to tailor interventions to individual immune vulnerabilities [181]. Such approaches may combine pharmacological modulation, dietary interventions, metabolic support, and lifestyle optimization to reshape immune trajectories. Although conceptually aligned with network-based models of immune aging, robust clinical validation and cost-effective implementation remain significant barriers [53,181,182,183,184,185].

Collectively, these diverse strategies underscore that immune aging is modifiable at multiple levels. While no single intervention will universally reverse immune aging, a multimodal approach that combines mechanistic pharmacology with lifestyle, nutrition, and microbiome targeting holds promise for attenuating immunosenescence, enhancing resolution, and ultimately reducing the burden of age-related diseases. Such multimodal strategies aim to target core inflammatory circuits, enhance regenerative capacity, restore systemic metabolic regulation, and address individualized immune vulnerabilities in an integrated and precision-oriented manner. Current evidence supports cautious optimism, but rigorous clinical trials, long-term safety assessments, and improved biomarker-guided patient selection are essential before widespread clinical implementation.

## 8. Systems-Level and Network Perspectives on Immune Aging

Immune aging is best understood not as a collection of isolated defects, but as a complex, interconnected reconfiguration of immune and tissue networks that alters how the body responds to internal and external stressors [186]. Aging causes coordinated changes in innate and adaptive immunity, metabolic pathways, and inter-organ communication, creating a web of interactions whose emergent properties differ fundamentally from those of younger systems. Modern multi-omics and deep immunophenotyping studies emphasize this network behavior, showing that age-associated immune changes involve coordinated shifts across cell subsets, cytokine profiles, and systemic signatures rather than single pathway perturbations [187].

**Figure 7:**
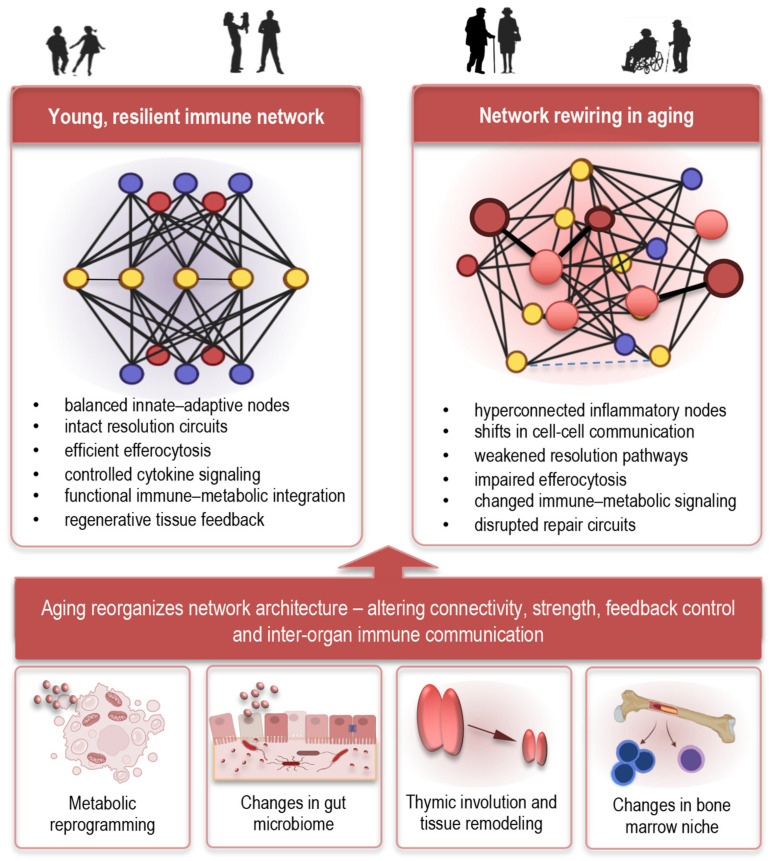
Systems-level network rewiring in the aging immune system. Aging reshapes immune system organization through coordinated alterations in cell–cell communication, signaling connectivity, immune–metabolic interactions, and resolution–regeneration feedback circuits. In youthful immune networks, balanced signaling and efficient resolution maintain tissue homeostasis. In aging, inflammatory nodes become hyperconnected, resolution and repair pathways weaken, and persistent damage-associated signals promote self-sustaining inflammatory loops. These network-level alterations contribute to chronic inflammation, impaired tissue regeneration, and the development of age-related pathologies.

Central to this perspective is the concept that immune resilience—the ability to mount effective responses and return to homeostasis—is a network property arising from interaction among cellular populations, signaling pathways, and tissue niches (Figure 7, left). With aging, network dynamics shift toward a state dominated by inflammaging and reduced responsiveness to perturbations [12] (Figure 7, right). Operationally, immune network rewiring can be inferred from shifts in cell–cell communication networks, altered cytokine interaction patterns, persistent inflammatory feedback loops, impaired resolution signaling, and changes in immune–metabolic regulatory nodes observed across aging tissues [188]. From a systems perspective, aging does not simply weaken immune function but reorganizes network architecture, altering connectivity strength, feedback control, and inter-organ immune communication [12,188].

Examples of experimentally observable network alterations include changes in cytokine co-expression patterns, shifts in immune cell interaction networks revealed by single-cell analyses, and altered resolution mediator signaling dynamics. Elements of this rewiring include persistent pro-inflammatory feedback loops, increased representation of regulatory and suppressive cell phenotypes, and altered communication between immune cells and tissues such as bone marrow, adipose, and the gut mucosa (Figure 7, bottom). These integrated changes impact not only host defense but also tissue maintenance, metabolic regulation, and repair processes, contributing to multisystem dysfunctions characteristic of ARD [12,25,73,158,187,188].

A critical feature emerging from systems analyses is the interdependence of metabolic and immune networks. Age-related metabolic shifts influence immune cell energy states, redox balance, and mediator synthesis, which in turn shape cytokine networks and cell–cell interactions. For example, dysregulated NF-κB and nutrient-sensing pathways such as mTOR and AMPK, detected across multiple immune subsets, can simultaneously affect inflammatory output and cellular metabolism, linking systemic metabolic aging with immune dysfunction [8]. Such network nodes serve as integration points where metabolic, inflammatory, and aging signals converge, highlighting opportunities for interventions that target foundational cross-talk rather than discrete pathways.

Systems approaches also underscore the role of tissue niches and inter-organ communication in immune aging. The gut microbiome, thymus, lymphoid architecture, and stromal environments each function as network hubs that influence systemic immune behavior. Aging-associated remodeling in these niches—such as microbiome dysbiosis and thymic involution—alters antigen exposure, cytokine gradients, and developmental signals, which ripple through the immune network and modulate disease susceptibility across organs [179].

Finally, systems-level frameworks help explain observed heterogeneity in aging trajectories. Integrative immune profiling reveals that individuals diverge in their immune network states, with some maintaining more resilient, youthful signatures while others show pronounced pro-inflammatory and dysregulated profiles even at similar chronological ages. These network signatures can predict differences in vaccine responses, frailty, and disease risk, providing a basis for precision approaches to modulate immune aging [12,53,73,187].

Together, these insights emphasize that immune aging is not the failure of a single cell type or pathway but a reprogramming of interacting immune and physiological networks. Understanding and intervening in these network dynamics may offer a path toward restoring immune balance, enhancing resilience, and mitigating the multi-organ impacts of aging.

## 9. Knowledge Gaps and Future Directions

Despite substantial progress in defining the cellular and molecular features of immune aging, major knowledge gaps remain that limit translation into effective interventions. A central unresolved issue is the causal hierarchy among immunosenescence, inflammaging, impaired resolution, and defective regeneration. While these processes clearly reinforce one another, the temporal sequence and dominant drivers likely vary across tissues and individuals, and remain poorly defined. Longitudinal human studies integrating immune, metabolic, and tissue-level data are still scarce, constraining our ability to distinguish primary mechanisms from downstream consequences.

Another major gap concerns the resolution phase of inflammation in aging. Although reduced pro-resolving capacity is increasingly recognized as a key determinant of chronic inflammation, the precise defects in resolution circuits—whether at the level of mediator biosynthesis, receptor signaling, cellular responsiveness, or tissue context—are incompletely characterized in humans. Moreover, resolution biology has largely been studied in acute inflammation models, and its role in chronic, low-grade inflammatory states typical of aging requires further mechanistic and clinical investigation.

The heterogeneity of immune aging represents both a challenge and an opportunity. Individuals of similar chronological age can exhibit markedly different immune network states, inflammatory burdens, and disease susceptibility. The determinants of this heterogeneity—including genetics, early-life exposures, microbiome composition, metabolic status, and environmental stressors—are not yet fully integrated into unified models of immune aging. Future studies must move beyond population averages toward stratified and personalized approaches that identify distinct immune aging trajectories.

At a methodological level, there remains a need for systems-level integration across scales. While multi-omics technologies have expanded rapidly, linking molecular signatures to functional immune behaviors, tissue outcomes, and clinical phenotypes remains difficult. Improved computational models, network-based analyses, and experimentally testable frameworks are required to translate complex datasets into actionable biological insight.

Finally, therapeutic development faces unresolved questions regarding timing, specificity, and safety. It is unclear when during the aging process immune-targeted interventions are most effective, whether treatments should aim to suppress inflammation, enhance resolution, restore immune diversity, or combine these strategies, and how to avoid compromising host defense. Addressing these gaps will be essential for designing interventions that improve healthspan without increasing vulnerability to infection or malignancy.

## 10. Conclusions

Immune aging is a central, systems-level process that shapes the trajectory of aging and the development of ARDs. Rather than reflecting isolated cellular defects, immunosenescence and inflammaging emerge from a coordinated reprogramming of immune networks, characterized by chronic inflammatory activation, impaired resolution, defective regeneration, and maladaptive immune–tissue feedback loops. These alterations compromise immune adaptability, disrupt tissue homeostasis, and promote progressive functional decline across multiple organs.

A key insight emerging from recent work is that aging-associated inflammation is not merely excessive, but incompletely resolved. Defects in efferocytosis, resolution signaling, and regenerative responses transform otherwise protective immune reactions into persistent drivers of tissue damage and fibrosis. This failure to terminate and repair inflammation provides a unifying mechanism linking immune aging to diverse ARDs, including cancer, neurodegeneration, and cardiometabolic disease.

Viewing immune aging through a system and network lens offers a powerful framework for understanding its complexity and for identifying intervention points with broad impact. Therapeutic strategies that restore immune balance—by enhancing resolution, modulating metabolic–immune cross-talk, reducing senescence-associated inflammation, and preserving tissue repair capacity—hold promise for altering aging trajectories and extending healthspan.

While immune aging also influences susceptibility to infection and vaccine responsiveness, the present review emphasizes chronic inflammatory and degenerative ARDs in which impaired resolution, immune–metabolic dysregulation, and defective regeneration represent central and therapeutically targetable mechanisms. Ultimately, targeting immune aging represents an opportunity not only to treat individual diseases, but to modify a fundamental biological process underlying multiple age-related pathologies. Continued integration of mechanistic biology, systems-level analysis, and carefully designed human studies will be essential to translate these insights into effective and safe interventions for aging populations.

## Data Availability

No new data were created or analyzed in this study.
